# Difference between medial and lateral tibia plateau in the coronal plane: importance of preoperative evaluation for medial unicompartmental knee arthroplasty

**DOI:** 10.1186/s12891-022-05298-6

**Published:** 2022-04-09

**Authors:** Sager h Alruwaili, Kwan Kyu Park, Ick Hwan Yang, Woo-Suk Lee, Byung-Woo Cho, Hyuck Min Kwon

**Affiliations:** 1 Department of Orthopedic Surgery, College of Medicine, Jouf University, Sakakah, Saudi Arabia; 2 Department of Orthopedic Surgery, College of Medicine, Yonsei University, Seoul, Korea; 3 Department of Orthopaedic Surgery, Yongin Severance Hospital, Yonsei University College of Medicine, 363, Dongbaekjukjeon-daero, Yongin, 16995 Gyeonggi-do Korea

**Keywords:** Medial unicompartmental knee arthroplasty, Knee osteoarthritis, Total knee replacement, Joint line orientation

## Abstract

**Background:**

Setting bone cutting levels for different joint line orientations of the medial and lateral tibia plateaus in individual patients is not clear. We aimed to evaluate the difference between joint line orientation of the medial and lateral tibia plateaus relative to the horizontal line of mechanical axis of tibia as tibial plateau difference (TPD) for an optimal tibial bone cut in medial unicompartmental knee arthroplasty (UKA) and determine which factors could influence TPD. We aimed to investigate the effect of preoperative TPD on polyethylene liner size in medial UKA.

**Methods:**

TPD in the coronal plane were measured in 181 female patients (181 knees). To determine the morphology of proximal tibia according to the severity of osteoarthritis, the patients were classified into three groups based on diagnosis and treatment: 80 who underwent robot-assisted medial UKA, 45 who underwent total knee arthroplasty (TKA), and 56 with early-stage osteoarthritis (OA) who had conservative management. Also, we divided the medial UKA group into two groups according to TPD (greater than or less than 5 mm) and compared polyethylene liner sizes.

**Results:**

No significant difference was observed in TPD (*p* = 0.662), difference between the medial and lateral femoral condyle levels (*p* = 0.54), medial proximal tibial angle (*p* = 0.169), or posterior tibial slope (*p* = 0.466) among the three groups. Increased TPD was significantly associated with increased mechanical femorotibial angle(mFTA) (*p* < 0.01). The medial UKA group was divided into two groups according to TPD greater or less than 5 mm. Thicker polyethylene liners were used for groups with TPD greater than 5 mm (8.5 ± 0.7 mm versus 8.2 ± 0.3 mm, *p* = 0.01). Additionally, the proportion of patients using the thinnest polyethylene (8 mm) in each TPD group (greater or less than 5 mm) was higher in patients with TPD less than 5 mm (82.4% versus 58.7%, *p* = 0.038).

**Conclusions:**

Preoperative measurement of TPD is important to help surgeons predict the most appropriate bone cutting level in the coronal plane in primary medial UKA. Tibial bone resection would be likely to be thicker than needed in patients with increased TPD in medial UKA.

## Background

Medial unicompartmental knee arthroplasty (UKA) is becoming common, especially among young patients or those with early localized osteoarthritis (OA) [1–4]. Compared to total knee arthroplasty (TKA), UKA is advantageous because it restores physiological knee kinematics, resulting in better postoperative function with preservation of the bone and ligaments and allowing for less invasive procedures when revision surgery is needed [5, 6]. Even though flexion and extension gap balancing is most important factor for surgical outcomes with medial UKA, minimal tibial resection is also important to sit on stronger subchondral bone and prevent excessive bone loss [7]. Several studies report that surgeons should preserve the joint line at the anatomical point in medial UKA surgery to determine the bone cutting level of the tibial plateau [8, 9]. However, no consensus exists regarding how much tibial bone cutting is appropriate for primary medial UKA.

Surgeons may follow the patient’s natural anatomy such as joint line orientation and level for bone cutting of the medial tibial plateau in medial UKA. If one patient shows a difference of only a few millimeters between joint line orientation of the medial and lateral tibial plateau relative to a line perpendicular to the mechanical axis of tibia in the coronal plane, while another shows a larger difference (e.g., 8–10 mm), surgeons may consider different cutting levels for these patients. An especially substantial difference is reported in the morphology of the tibia plateau and common proximal tibia vara in knee osteoarthritic patients who are Asian compared to Western populations [10, 11]. The tibia bone cutting level in medial UKA could have significant effects on surgical outcomes of implant fixation and ligament balance. Nevertheless, how to set bone cutting levels for different joint line orientation of medial and lateral tibia plateaus level by patient is not known. Thus, we hypothesized that differences in medial and lateral tibia plateau joint line orientations of patients would affect tibial bone cutting levels in medial UKA and be associated with polyethylene liner size for ligament balancing.

Therefore, the purpose of this study was to evaluate the differences in medial and lateral tibia plateaus joint line orientations for optimal tibial bone cutting in medial UKA and to identify factors influencing tibia plateau differences. We aimed to investigate the effect of differences in joint line orientation of medial and lateral tibia plateaus on polyethylene liner size.

## Methods

### Subjects

This retrospective study analyzed 181 female patients (181 knees) who were consecutively treated between January 2016 and January 2018. Patients were distributed among three groups based on diagnosis and management: Group 1 comprised 45 patients with severe degenerative medial OA (Kellgren-Lawrence grade III or IV) of the knee who were scheduled for TKA. Group 2 included 80 patients with severe degenerative isolated medial compartment OA (Kellgren-Lawrence grade III or IV) of the knee who were scheduled for robotic-assisted medial UKA (MAKO Surgical Corporation, Fort Lauderdale, FL, USA). After MRI evaluation, patients without lateral compartment OA or patellofemoral and without ACL deficiency were enrolled in the medial UKA group. Group 3 comprised 56 patients with early stage medial compartment OA Kellgren-Lawrence grade I or II) of the knee who received conservative management at an outpatient clinic. Patients with lower-extremity fixed deformities such as severe varus or a valgus knee deformity of greater than 10 degrees, inflammatory arthritis, post-traumatic arthritis, or a history of previous surgery including arthroscopic procedures were excluded.

### Data collection

**Fig. 1:**
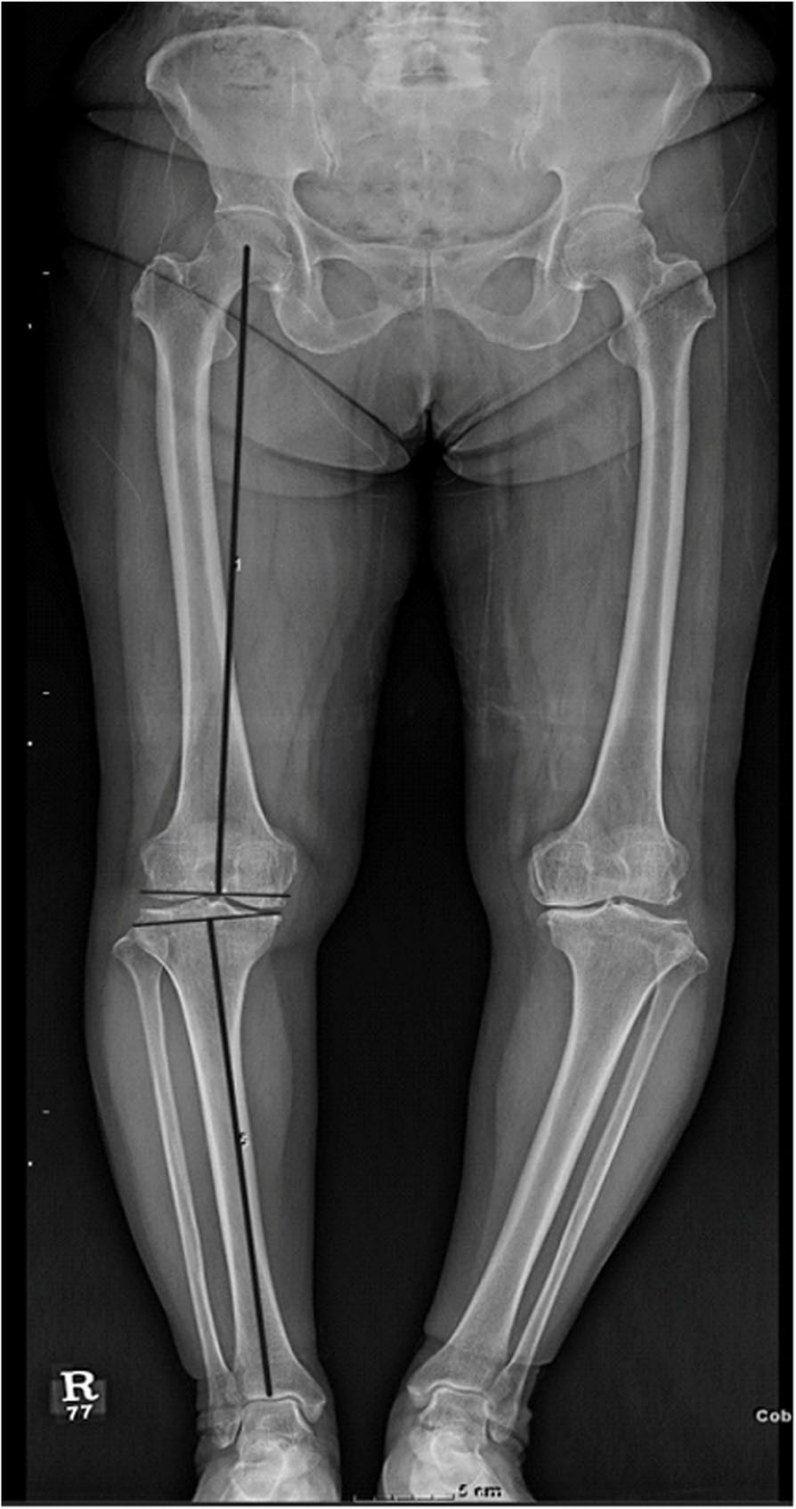
Measurement of the mFTA with two perpendicular lines at the level of the femoral intercondylar notch and at the level of the medial tibia plateau mFTA, mechanical femoro-tibial angle

**Fig. 2:**
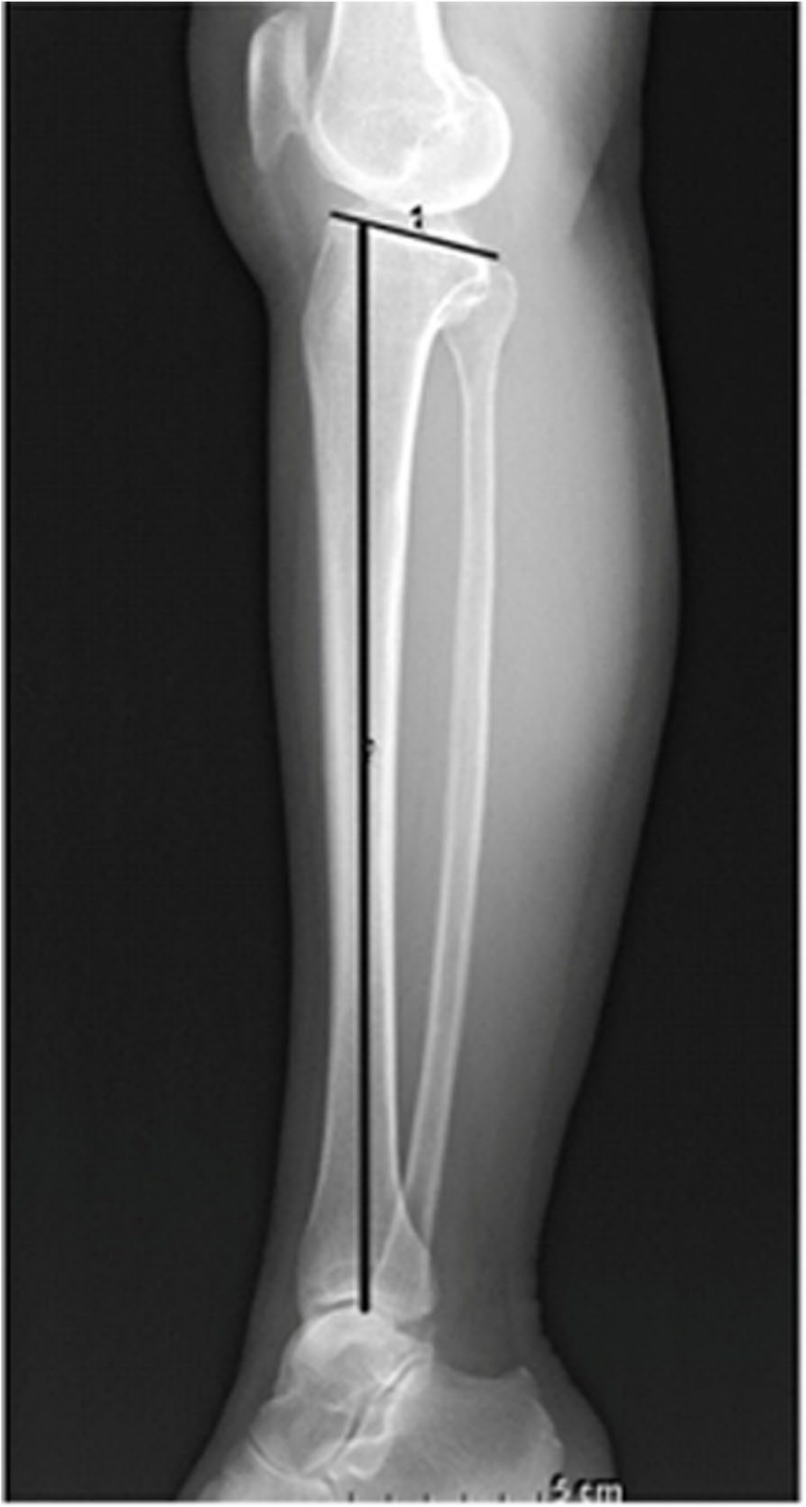
Measurement of the posterior tibial slope, defined as the angle between a line perpendicular to the long axis of the tibia and a line from the anterior to posterior medial tibial plateau

**Fig. 3:**
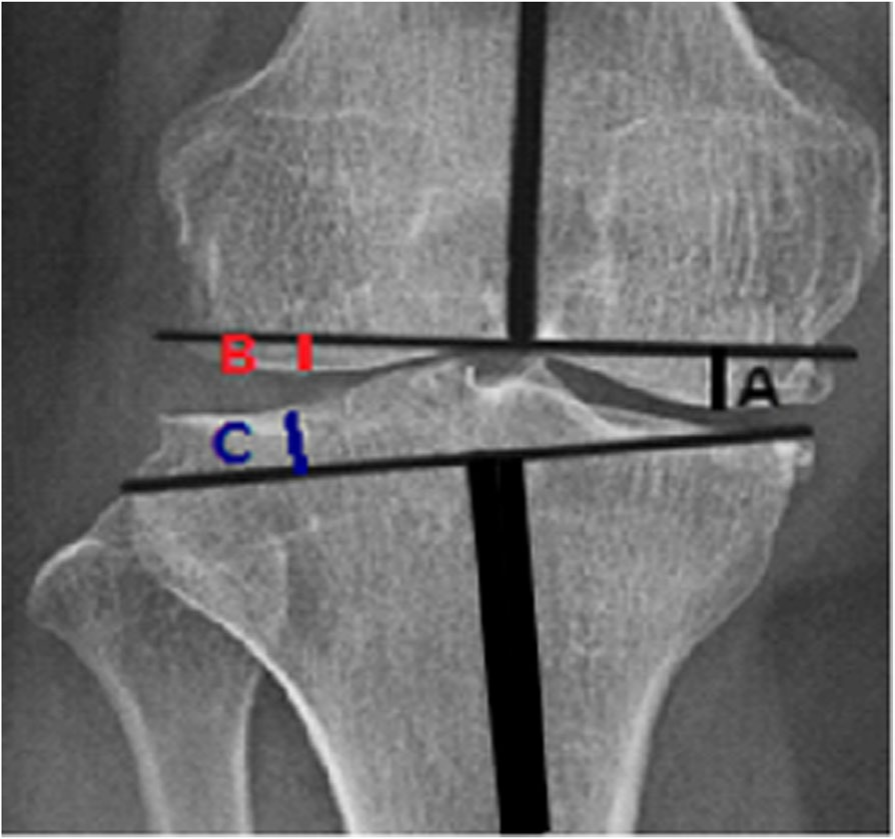
Measurement of TPD and FD. After the mFTA was measured, a perpendicular line was drawn at the level of the intercondylar notch with two perpendicular lines drawn from this line to the medial femoral condyle joint surface (line A) and lateral femoral condyle joint surface (line B). The difference between the two lines (A-B) was the FD. On the tibial side, the TPD was measured by drawing a perpendicular line on the mechanical axis of the tibia starting from the medial tibial plateau joint surface. The perpendicular distance from this line to the lateral tibial plateau was the TPD (line C). mFTA, mechanical femoro-tibial angle; FD, the difference between the medial and lateral femoral condyle levels; TPD, tibia plateau difference

Several variables were measured on long bone radiographs of patients taken by a standard radiographic protocol using a digital radiographic system (Definium8000, GE Healthcare, Waukesha, WI, USA). All radiographs were taken at first outpatient clinic visit with true anterior–posterior radiographic images with the patella as the center. The mechanical femoro-tibial axis (mFTA), medial proximal tibial angle (MPTA), tibial plateau difference (TPD, tibial plateau difference; difference between joint line orientation of the medial and lateral tibia plateaus relative to the horizontal line of mechanical axis of tibia in the coronal plane), and difference between the medial and lateral femoral condyles in the coronal plate (FD) were measured on the anterolateral view. The posterior tibial slope was measured on the lateral view (Figs. 1 and 2). The mTFA was measured as the angle between the line from the center of the femoral head to the center of the knee, and the line from the center of the knee to the center of the ankle (Fig. 1). The medial proximal tibial angle was measured as the angle between the tibial mechanical axis and the articular surface of the proximal tibia. TPD was measured by drawing a perpendicular line on the mechanical axis of the tibia starting from the most distal point of the medial tibial plateau. The perpendicular distance from this line to the joint surface on the lateral tibia plateau was the TPD (Fig. 3). The FD was defined as the difference between two lines that started from a perpendicular line at the level of the intercondylar notch of the mechanical axis of the femur to the medial and lateral joint surfaces of the femoral condyles (Fig. 3).

On the true lateral knee radiograph, the posterior tibial slope was measured as the angle between a line perpendicular to the long axis of the tibia and a line from the anterior to the posterior medial tibial plateau (Fig. 2). All measurements were taken by two orthopedic surgeons and repeated after an interval of more than 2 weeks. The degree of measurement reliability was evaluated using intraclass correlation coefficients (ICC). Calculation of ICC values was performed by two experienced orthopedic surgeons. For ICC analysis, values less than 0.2 were considered to indicate poor agreement; 0.21 to 0.40, fair agreement; 0.41 to 0.60, moderate agreement; 0.61 to 0.80, good agreement; and above 0.80, excellent agreement [12].

### Surgical procedures

All TKA and medial UKA surgeries for Group 1 and Group 2 patients were performed by a single experienced surgeon (KKP) using a parapatellar approach. All patients in Group 1 (TKA) received TKA with posterior stabilized prosthesis (Zimmer LPS-Flex Gender Knee, Zimmer, Inc., Warsaw, IN, USA) using a fixed-bearing polyethylene liner. All patients in Group 2 received robotic-assisted medial UKA with RESTORIS MCK implant (Stryker Corp., Mahwah, NJ, USA). In patients with robotic-assisted medial UKA, preoperative CT scans were performed to facilitate preoperative surgical planning. Intraoperative registration and ligament balancing were performed by recording the flexion and extension gaps through the knee’s range of motion. Minimal bone resection was performed using a high-speed burr with haptic guided robotic-arm to preserve the native soft tissue balance of the knee and to achieve minimal extension gap and insert a thinner polyethylene liner. The posterior slope of the proximal tibia resection was set to make a 5˚ posterior slope in the sagittal plane*.* The polyethylene liner sizes of medial UKA patients (Group 2) were compared according to TPD on preoperative long bone radiographs.

### Statistical analysis

All analyses were performed with the statistical program SPSS, version 23 (IBM SPSS statistics, USA). Kolmogorov–Smirnov tests revealed that all variables were normally distributed. The mean and standard deviation of continuous variables were recorded for each group (TKA, medial UKA, and early stage OA group). ANOVA and Student’s t-test were used for comparisons among groups. Multiple linear regression analysis was performed to identify factors influencing TPD. Subgroup correlation analysis between TPD and other variables was also performed for each group. The *P* value for statistical significance was set at 0.05. The statistical software G*Power (version 3.1.9.4; Hein-rich Heine Universität Düsseldorf, DE) was used for sample size calculation. A total of 42 subjects were required to perform ANOVA analysis with a power of 0.8, effect size of 0.5, and alpha error of 0.05, and the sample size of this study satisfied this requirement.

## Results

Demographic data and radiographic parameters for the three groups are in Table 1. Group 1 (TKA) had a larger mFTA (6° ± 3.8°; range, 0°-10°) than Group 2 (medial UKA) (4.8° ± 3.1°; range, 0°-8°) (*p* < 0.01) and Group 3 (early stage OA) (4.6° ± 2.6°; range, 0°-9°) (*p* < 0.01). Patients in the medial UKA and early stage OA groups did not differ in mFTA. Most patients in Group 1 (TKA) had mFTA 5°-10° (75.6%) for varus angulation. Most patients in Group 2 (medial UKA) (62.5%) had mFTA 5°-10°, followed by 0°-5° (37.5%) for varus angulation. Most patients in Group 3 (early stage OA) had mFTA 0°-5° (57.1%) (Table 1).

**Table 1:** Comparison of descriptive data among the three groups

	**TKA** **(*n* = 45)**	**Medial UKA** **(*n* = 80)**	**Early stage OA** **(*n* = 56)**	*p*	p*	*p†*	*p‡*
**Age (years)**	71.9 ± 6.1 (52–86)	64.8 ± 6.6 (50–81)	67.7 ± 5.8 (60–84)	< 0.01	< 0.01	< 0.01	< 0.01
**BMI** **(Kg/m**^**2**^**)**	25.6 ± 2.3 (18.1–29.7)	25.2 ± 2.3 (20.6–30.3)	24.7 ± 3 (19.9–36)	0.345	0.918	0.367	1.000
**mFTA(°)**	6 ± 3.8 )0–10)	4.8 ± 3.1 (0–8)	4.6 ± 2.6 (0–9)	< 0.01	< 0.01	< 0.01	1.000
**0° to 5°**	11 (24.4%)	30 (37.5%)	32 (57.1%)				
**5° to 10°**	34 (75.6%)	50 (62.5%)	24 (42.9%)				
**MPTA (°)**	86.2 ± 1.9 (84–90)	86.3 ± 1.7 (84–90)	86.8 ± 1.6 (82–90)	0.769	0.887	0.466	0.366
**TPD (mm)**	5.2 ± 2.8 (-3–9.4)	5.9 ± 2.2 (-1.5–9.8)	5.6 ± 2.4 (-2.8- 9.2)	0.882	0.949	0.883	0.972
**Posterior tibia slope(°)**	10.5 ± 3 (-2.2–15.3)	9.8 ± 2.7 (4.4–16.3)	10.7 ± 2.7 (4.9–16.6)	0.066			
**FD (mm)**	1 ± 1.4 (-1.7- 3.9)	1.3 ± 1.3 (-1.5–4.3)	1.1 ± 1.5 (-1.5–4.4)	0.74	0.982	0.643	0.457

Data are mean ± standard deviation (range)

*TKA* total knee arthroplasty, *UKA* unicompartmental knee arthroplasty, *OA* osteoarthritis, *mFTA* medial proximal tibial angle, *MPTA* mechanical femoro-tibial axis, *TPD* tibia plateau difference, *FD* difference between the medial and lateral femoral condyle levels

p* p-value between TKA and medial UKA group, *p*† p-value between TKA and early stage OA group, *p‡* p-value between medial UKA and early stage OA group

No significant difference was observed in BMI (*p* = 0.345), TPD (*p* = 0.882), FD (*p* = 0.74), MPTA (*p* = 0.769), or posterior tibial slope (*p* = 0.066) among the three groups (Table 1). In all groups, most patients had TPD 5–10 mm. No patients had TPD greater than 10 mm (Table 2). Minimal bone cutting aimed to balance flexion and extension gaps and ligaments during robotic-assisted medial UKA. When Group 2 (medial UKA) was divided into two groups according to TPD (greater than and less than 5 mm), thicker polyethylene liners were used for the group with greater than 5 mm (8.5 ± 0.7 mm versus 8.2 ± 0.3 mm, *p* = 0.01). The proportion of patients using the thinnest polyethylene in each TPD group (greater than and less than 5 mm) was higher in patients with TPD less than 5 mm (82.4% versus 58.7%, *p* = 0.038) (Table 3).

**Table 2:** TPD distribution in the three groups of patients

**TPD (mm)**	**Medial UKA** **(*n* = 80)**	**TKA** **(*n* = 45)**	**Early stage OA** **(*n* = 56)**
**-3 to 0**	4 (5%)	2 (4.4%)	2 (3.6%)
**0 to 5**	30 (37.5%)	18 (40%)	18 (32.1%)
**5 to 10**	46 (57.5%)	25 (55.6%)	36 (64.3%)

*TKA* total knee arthroplasty, *UKA* unicompartmental knee arthroplasty, *OA* osteoarthritis, *mFTA* mechanical femoro-tibial axis, *TPD* tibia plateau difference, *FD* difference between the medial and lateral femoral condyle levels

**Table 3:** Comparison of the greater TPD and lesser TPD groups in medial UKA

	**TPD < 5 mm (*N* = 34)**	**TPD 5–10 mm (*N* = 46)**	***p***
**Age (years)**	62.6 ± 6.2	64.6 ± 5.6	0.195
**BMI (Kg/m**^**2**^**)**	24.7 ± 2.7	25.4 ± 2.0	0.181
**mFTA (°)**	4.9 ± 3.6	5.1 ± 4.1	0.818
**Posterior tibia slope (°)**	7.6 ± 3.1	8.6 ± 3.0	0.153
**Polyethylene size (mm)**	8.2 ± 0.3	8.5 ± 0.7	0.01
**Polyethylene size distribution**			
** 8 mm**	28	27	
** 9 mm**	6	14	
** 10 mm**	0	5	
**Proportion of thinnest PE (8 mm)**	28/34 (82.4%)	27/46 (58.7%)	0.038

Data are mean ± standard deviation (range)

*UKA* unicompartmental knee arthroplasty, *BMI* body mass index, *mFTA* mechanical femoro-tibial angle, *TPD* tibia plateau difference

Multiple linear regression analysis was performed to determine factors influencing TPD in all patients. Multiple linear regression analysis revealed that TPD was significantly influenced by increased mFTA (*p* < 0.01; beta coefficients = 0.333) and decreased FD (*p* = 0.017; beta coefficient = 0.341) but not BMI (*p* = 0.110), age (*p* = 0.601), or posterior tibial slope (*p* = 0.398) (Table 4).

**Table 4:** Multiple linear regression analysis of factors influencing tibial plateau difference (R-square = 0.192)

	**Beta coefficients**	**95% CI**	***p***
Age	0.016	-0.044 -0 .075	0.601
BMI	-0.088	-0.238—0.061	0.110
mFTA	0.333	0.214—0.451	< 0.01
Posterior tibia slope	0.059	-0.079—0.198	0.398
FD	0.341	0.062 – 0.621	0.017

*95% CI* 95% confidence interval, *mFTA* mechanical femoro-tibial angle, *TPD* tibia plateau difference, *FD* difference between medial and lateral femoral condyle level

Table 5 shows the ICC values for intra-and interobserver variability for all radiologic measurements. All of the ICC values for both intraobserver reliability and interobserver reliability were greater than 0.9.

**Table 5:** Intraclass correlation coefficient values of all radiologic measurements for intra-and interobserver variability

**Radiologic parameter**	**ICC**	
	**Intraobserver**	**Interobserver**
mFTA	0.95 (0.93–0.96)	0.94 (0.93–0.95)
MPTA	0.98 (0.96–0.99)	0.96 (0.94–0.98)
TPD	0.93 (0.91–0.96)	0.96 (0.95–0.97)
Posterior tibial slope	0.91 (0.88–0.94)	0.90 (0.89–0.92)
FD	0.91 (0.89–0.92)	0.95 (0.93–0.97)

*ICC* intraclass correlation coefficient, *mFTA* medial proximal tibial angle, *MPTA* mechanical femoro-tibial axis, *TPD* tibia plateau difference, *FD* difference between the medial and lateral femoral condyle levels

## Discussion

The most important finding of this study is that increased preoperative TPD of patients receiving robotic-assisted medial UKA was significantly associated with preoperative increased mFTA. Increased mFTA in varus knees increased total wear on the medial tibia plateau (as increased TPD) and the medial femoral condyle (as decreased FD), which supports load on the mechanical axis. Even though TPD was affected by the degree of varus angulation in our study, no significant difference was seen in TPD among early stage OA, medial UKA, and TKA groups. Furthermore, increased preoperative TPD greater than 5 mm with robotic-assisted medial UKA was associated with requiring thicker PE. This suggests that more tibial bone cutting may be needed for ligament balance in patients with increased TPD in medial UKA. The use of thin polyethylene liners for medial UKA is important because it has demonstrated excellent long-term clinical outcomes [13] and is influenced by the tibia bone cutting level. Although each implant manufacturer has a recommended cutting-level guide for the proximal tibia for medial UKA, depending on implant characteristics, no consensus exists on the ideal amount of proximal bone resection from the tibia. Minimizing tibia resection while considering preservation of the joint line and equivalence of the flexion and extension gaps is known to be the most important factor for survival after medial UKA [9, 14, 15]. However, little is known about the relationship between proper bone resection and the shape of the medial and lateral tibia plateaus. The amount of tibial resection in medial UKA depends on surgeon experience and intraoperative decisions, and has not been well studied for patients with OA. In this retrospective study, we found a significant relationship between mFTA and differences in the medial and lateral tibial plateau levels. We might need to consider tibial cutting level in medial UKA depending on the shape of the patient’s tibia plateau and difference between joint line orientation of medial and lateral plateaus.

Correcting limb alignment to neutral or slight varus has been advocated in the literature [16, 17]. Hernigou et al. studied the effects of knee alignment after medial UKA on polyethylene wear and lateral compartment degeneration. They found that undercorrection increases polyethylene wear while overcorrection to valgus increases lateral compartment degeneration [18]. Some authors studied the predicted amount of varus alignment correction and the amount of joint line elevation in medial UKA [19]. They found a significant correlation between joint line elevation and amount of limb correction. The amount of limb alignment correction was determined by the amount of tibial bone cut and the size of polyethylene used. Although we performed robotic-assisted medial UKA to obtain proper ligament balance and to minimize bone cutting using high-speed burr and a thinner PE target (8 mm), 27 patients (58.7%) in the higher TPD group (greater than 5 mm) needed 8-mm PE, whereas 28 patients (82.4%) in the lower TPD group (less than 5 mm) needed 8-mm PE. The need for a thicker polyethylene liner could result from excessive tibia bone cutting level or injury to or attenuation of the medial collateral ligament [13]. Because we confirmed that patients with increased preoperative TPD in medial UKA required thicker PE, we believe that decisions about the cutting level of the proximal tibia should be made based on patient anatomy and deformity and that measuring mFTA and TPD would be helpful to determine how much bony cutting is needed in medial UKA to achieve good long-term results.

The main concern for surgeons in revising medial UKA to TKA is the need for augments, a stem, or both due to aseptic loosening, osteoarthritis progression, and unexplained pain [20]. Although the use of PE thickness as a surrogate for tibial bone loss is controversial because of assumptions about preservation of the joint line and adequate ligament balance [21], we performed medial UKA using a robotic-assisted system with accurate preservation of joint line and adequate ligament balance. Reducing proximal tibial bone cutting in patients with a larger TPD in primary medial UKA and using thinner PE liners for balancing might reduce the chance that more aggressive implants would be needed on the tibial side, such as a wedge augment or long stem, during revision surgery. Therefore, evaluating preoperative TPD in medial UKA would help minimize bone defects that may occur in revision surgery by performing appropriate tibia bone cutting.

This study had some limitations. First, all patients in this study were female. Female gender dominance has been found in most TKA studies [22], and is particularly remarkable among patients with knee OA in Korea [23, 24]. Care should be taken when applying our results to males or other ethnic groups. Second, this was a retrospective study that did not assess postoperative outcomes. We focused on the preoperative radiographs of medial UKA, TKA, and early stage OA patients, so further prospective studies that assess different cutting levels of the proximal tibia in medial UKA, polyethylene insert thickness, and postoperative alignment would help with preoperative planning for UKA. In addition, since we analyzed only the coronal plane of radiographs, not MRI, cartilage thickness was not reflected. Also, a comprehensive analysis with sagittal plane radiographs was not performed, this was also a limitation of this study. Finally, we analyzed only a limited number of patients, so further studies are needed that include more patients and assess more variables. Additionally, because bone resection was performed with a high-speed burr in UKA of this study, information on the thickness of the tibia bone cutting surface could not be obtained. Also soft tissue balancing was achieved by minimizing soft tissue release to preserve the native soft tissue, therefore accurate and objective information about soft tissue balancing could not be obtained. Nevertheless, this study evaluated the importance of preoperative measurement of TPD and its clinical significance for the coronal position of the tibial component in medial UKA.

## Conclusions

A significant association was observed between joint line orientation of medial and lateral tibial plateaus and mFTA in preoperative evaluation. Preoperative planning is key to the success of primary medial UKA and revision surgery, so preoperative measurement of TPD and mFTA is important to help surgeons predict the most appropriate bone cutting level and position of the tibial component in the coronal plane in primary medial UKA. Tibial bone resection would be likely to be thicker than needed in patients with increased TPD in medial UKA, so care should be taken to prevent excessive bone loss in these patients.

## Data Availability

The datasets generated and analyzed during the current study are not publicly available due to privacy concern of participants but are available from the corresponding author on reasonable request.

## Abbreviations

TPD: Tibial Plateau Difference
UKA: Unicompartmental Knee Arthroplasty
TKA: Total Knee Arthroplasty
OA: Osteoarthritis
mTFA: Mechanical femoro-tibial axis
MPTA: Medial proximal tibial angle
